# An AIEgen/graphene oxide nanocomposite (AIEgen@GO)‐based two‐stage “turn‐on” nucleic acid biosensor for rapid detection of SARS‐CoV‐2 viral sequence

**DOI:** 10.1002/agt2.195

**Published:** 2022-04-11

**Authors:** Qin Zhang, Bohan Yin, Jianhua Hao, Linjie Ma, Yingying Huang, Xueying Shao, Chuanqi Li, Zhiqin Chu, Changqing Yi, Siu Hong Dexter Wong, Mo Yang

**Affiliations:** ^1^ Department of Biomedical Engineering The Hong Kong Polytechnic University Hong Kong China; ^2^ Department of Applied Physics The Hong Kong Polytechnic University Hong Kong China; ^3^ Department of Electrical and Electronic Engineering Joint Appointment with School of Biomedical Sciences The University of Hong Kong Hong Kong China; ^4^ Key Laboratory of Sensing Technology and Biomedical Instruments (Guangdong Province) School of Biomedical Engineering Sun Yat‐Sen University Guangzhou PR China

**Keywords:** aggregation‐induced emission (AIE) luminogen, graphene oxide, SARS‐CoV‐2 detection

## Abstract

The ongoing outbreak of Severe Acute Respiratory Syndrome Coronavirus 2 (SARS‐CoV‐2) pandemic has posed significant challenges in early viral diagnosis. Hence, it is urgently desirable to develop a rapid, inexpensive, and sensitive method to aid point‐of‐care SARS‐CoV‐2 detection. In this work, we report a highly sequence‐specific biosensor based on nanocomposites with aggregation‐induced emission luminogens (AIEgen)‐labeled oligonucleotide probes on graphene oxide nanosheets (AIEgen@GO) for one step‐detection of SARS‐CoV‐2‐specific nucleic acid sequences (*Orf1ab* or *N* genes). A dual “turn‐on” mechanism based on AIEgen@GO was established for viral nucleic acids detection. Here, the first‐stage fluorescence recovery was due to dissociation of the AIEgen from GO surface in the presence of target viral nucleic acid, and the second‐stage enhancement of AIE‐based fluorescent signal was due to the formation of a nucleic acid duplex to restrict the intramolecular rotation of the AIEgen. Furthermore, the feasibility of our platform for diagnostic application was demonstrated by detecting SARS‐CoV‐2 virus plasmids containing both *Orf1ab* and *N* genes with rapid detection around 1 h and good sensitivity at pM level without amplification. Our platform shows great promise in assisting the initial rapid detection of the SARS‐CoV‐2 nucleic acid sequence before utilizing quantitative reverse transcription‐polymerase chain reaction for second confirmation.

## Graphical abstract

An AIEgen‐graphene oxide (GO) nanocomposite‐based assay is designed for rapid detection of SARS‐CoV‐2 nucleic acids. The sensing mechanism is based on two‐stage fluorescence signal recovery due to fluorescence resonance energy transfer (FRET) effect by detaching AIEgen from GO surface and restricted intramolecular rotation (RIR) effect by formation of nucleic acid duplexes.

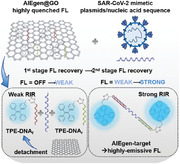

## 1 INTRODUCTION

The current Severe Acute Respiratory Syndrome Coronavirus 2 (SARS‐CoV‐2) outbreak, that causes Coronavirus Disease 2019 (COVID‐19), has rapidly evolved into a worldwide pandemic.^[^ ^1^, ^2^ ^]^ As of December 2021, there have been more than 281 million COVID‐19 confirmed cases, including over 5.4 million confirmed deaths reported worldwide, despite the asymptomatic cases.^[^ ^3^ ^]^ Moreover, many countries have implemented lockdown policies in multiple cities with high levels of COVID‐19 infection to prevent disease outbreaks. This pandemic has negatively affected both worldwide public health and the economy. Therefore, there is a vast demand for rapid, sensitive, and inexpensive diagnostic methods to identify infected persons from healthy individuals.

Nucleic acid diagnostic techniques, such as quantitative reverse transcription‐polymerase chain reaction (RT‐qPCR), remain the gold‐standard technique for the SARS‐CoV‐2 viral ribonucleic acid (RNA) detection with high sensitivity and accuracy.^[^ ^4^ ^]^ However, the lack of access to RT‐qPCR reagents, instruments, and professionally trained operators hampers the application of this technique, especially in resource‐limited developing countries.^[^ ^5^ ^]^ Despite the lengthy process, RT‐qPCR also requires a clean environment and a contamination‐free central laboratory for successful nucleic acid amplification, imposing an economic burden on society for collecting and analyzing samples. In addition, immunodiagnostic methods such as lateral flow immunoassay are easy to operate and allow rapid screening of SARS‐CoV‐2 antibodies within 15 min, but there may be problems with a potential false‐negative rate in the early stage of SARS‐CoV‐2 infection onset.^[^ ^6^ ^]^ To address the challenge of rapid outbreak of COVID‐19, it is highly desirable to develop a rapid point‐of‐care (POC) community‐level COVID‐19 testing kit as an initial screening of positive cases before utilizing RT‐qPCR for second confirmation.

Förster resonance energy transfer (FRET) biosensors that rely on nonradiative energy transfer from a donor to an acceptor, have been widely used in biomedical applications due to their high sensitivity and simple operation.^[^ ^7^, ^8^ ^]^ The sensing performance of such FRET‐based biosensors is mainly dependent on the design of donor and acceptor pairs. Recently, two‐dimensional (2D) nanomaterials, such as graphene oxide (GO),^[^ ^9^, ^10^, ^11^ ^]^ molybdenum disulfide (MoS_2_),^[^ ^12^, ^13^ ^]^ and metal organic framework,^[^ ^14^, ^15^ ^]^ have been used in biosensing areas due to their unique mechanical, optical, and electrical properties. Especially, these 2D nanomaterials can be utilized as excellent fluorescence quenchers (acceptor motif) in the construction of FRET‐based biosensor for nucleic acids detection due to their large surface area, high affinity to biomolecules and high quenching capability.^[^ ^9^, ^12^, ^16^, ^17^ ^]^ These properties enable the adsorption of fluorophore‐labeled single‐stranded deoxyribonucleic acid (ssDNA) probes (donor motif) on their surfaces to quench the basal fluorescence and thus optimize the signal‐to‐noise ratio. We have previously reported several sensing systems based on FRET mechanism for detecting different sequence‐specific nucleic acids, including tumor microRNA and viral genes.^[^ ^16^, ^17^, ^18^ ^]^ Generally, the sensing read‐out of these FRET biosensors often depends on the signal recovery of the fluorophores with the increase of the target concentrations, that is, the higher the concentration, the stronger the fluorescent signal until its saturation. However, many traditional fluorophores suffer from the aggregation‐caused quenching (ACQ) effect and can be quenched at high concentrations, which limits their sensitivity and detection range.^[^ ^19^ ^]^ Hence, it is necessary to explore an alternative strategy to overcome this limitation for optimizing the fluorescent read‐out of FRET biosensors.

The aggregation‐induced emission (AIE) fluorogen (AIEgen) is the most successful breakthrough against ACQ and photobleaching among different attempts.^[^ ^20^, ^21^ ^]^ AIEgens remain nonemissive in a well‐dissolved and dispersed state but become highly fluorescent‐emissive in an aggregated state. This phenomenon is theoretically due to the restriction of intramolecular rotation (RIR) that halts the nonradiative deactivation pathway and thus promotes light‐stimulated molecular excited states.^[^ ^22^ ^]^ Hence, it becomes possible to intensify the fluorescent signals even at a high fluorophore concentration (aggregated states) to increase the biosensor sensitivity. Tetraphenylethene (TPE) and its derivatives are well‐known and promising AIEgens as supersensitive fluorescent indicators for particular small molecules,^[^ ^23^, ^24^ ^]^ metal ions,^[^ ^25^, ^26^ ^]^ proteins,^[^ ^27^, ^28^ ^]^ and cellular membrane receptors.^[^ ^29^, ^30^ ^]^ These “turn‐on” processes can be achieved by forming intermolecular interactions (specific or nonspecific) between AIEgens and target molecules that induce RIR or self‐aggregation triggered by the decrease of solubility in an aqueous solution. Specifically, oligonucleotide‐conjugated TPE (TPE‐DNA)‐based AIE probes are of great interest in nucleic acid detection because of their good water solubility and the ability to form a nucleic acid duplex structure or even quadruplex conformation with the target sequence to activate RIR.^[^ ^31^, ^32^, ^33^ ^]^ Nonetheless, direct insertion of a sensing site into TPE molecules to build sensing nanoprobes can inevitably introduce a small amount of RIR, leading to a certain fluorescence background, thereby decreasing the signal‐to‐background ratio upon DNA hybridization. Therefore, engineering a cost‐effective AIEgen‐based biosensor with high sensitivity and low background is prudent to facilitate SARS‐CoV‐2 detection in settings with limited resources.

**FIGURE 1:**
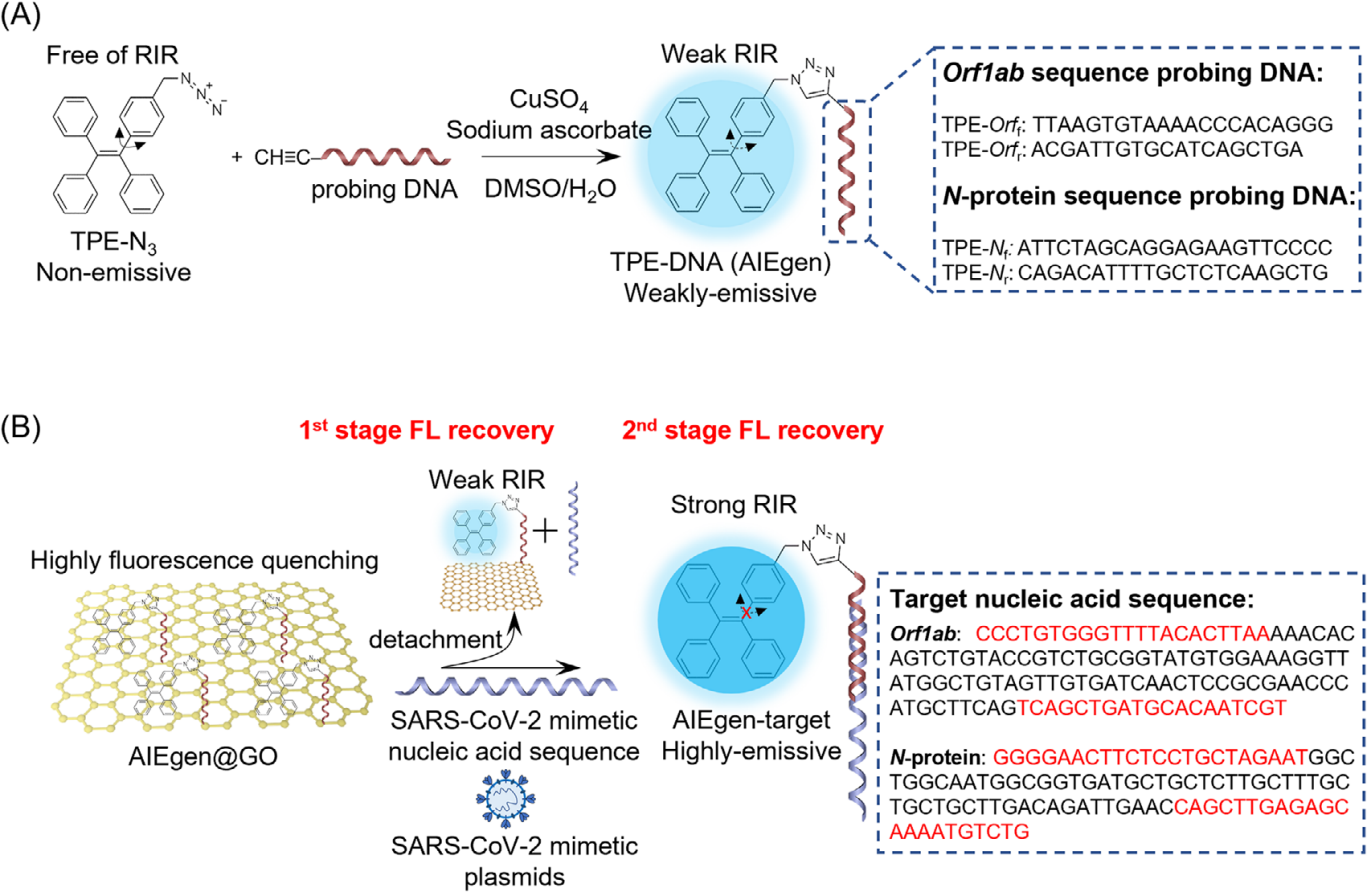
Schematic illustrations of synthesis and application of AIEgen/graphene oxide (AIEgen@GO) for rapid detection of Severe Acute Respiratory Syndrome Coronavirus 2 (SARS‐CoV‐2). (A) Synthetic route to tetraphenylethene (TPE)‐DNA (AIEgen) via a Cu(I) catalyzed click reaction. Primers of *Orf1ab* or *N* gene were selected as the probing sequences. (B) Coupling of AIEgen onto graphene oxide (GO) for quenching the pre‐existing weak restricted intramolecular rotation (RIR)‐induced fluorescence and enabling a dual entity of fluorescent amplification upon the sensation of the target sequence

In this study, we report a two‐stage “turn‐on” nucleic acid biosensing platform for rapid detection of SARS‐CoV‐2 viral sequence‐based on TPE‐DNA‐immobilized GO nanocomposite (AIEgen@GO) (Figure 1A). In the presence of target SARS‐CoV‐2 viral sequence, AIEgen on GO surface hybridizes with target viral sequence to form DNA/RNA duplex‐TPE molecules, which dissociate from the GO surface due to the weakening of the interactions between AIEgen and GO. Here, the detachment of AIEgen from GO surface increases the distance between AIEgen and GO, leading to the 1st stage of fluorescence recovery from “**OFF**” to “**WEAK**.” Moreover, the formation of nucleic acid duplexes restricts the intramolecular rotation of the phenylene ring in the TPE structure, mainly due to the increased rigidity and mass changes from ssDNA to dsDNA, leading to the further enhancement of the fluorescence recovery signal from “**WEAK**” to “**STRONG**” (Figure 1B).^[^ ^32^, ^33^ ^]^ GO (acceptor) plays a critical role in reducing the basal fluorescent level of TPE‐DNA (donor), and the formation of duplex‐TPE enhances the final fluorescence sensing signals. This one‐step sensation of the target nucleic acids triggers dual entities that make our AIEgen@GO biosensor unique from the existing fluorescent biosensors, which typically possess only a single fluorescent enhancement or single quenching entity, respectively (Table S1).^[^ ^34^, ^35^, ^36^, ^37^ ^]^ Using this probe, we demonstrate the rapid and specific detection of dual SARS‐CoV‐2 nucleic acid sequences of *Orf1ab* or *N* genes without amplification steps. Finally, SARS‐CoV‐2 plasmids are used to mimic real virus samples for testing the sensing performance of AIEgen@GO nanoprobes. The generation of SARS‐CoV‐2 plasmids by reverse genetics could bypass the limited availability of virus isolates for developing diagnostic tools, which are emerging as alternatives to real clinical samples.^[^ ^38^, ^39^, ^40^, ^41^ ^]^ The feasibility of our nano‐sensing platform for potential clinical application is demonstrated using SARS‐CoV‐2 plasmids with rapid detection around 1 h and good sensitivity at pM level without amplification.

## 2 RESULTS AND DISCUSSION

### 2.1 Synthesis and characterization of AIEgen@GO

**FIGURE 2:**
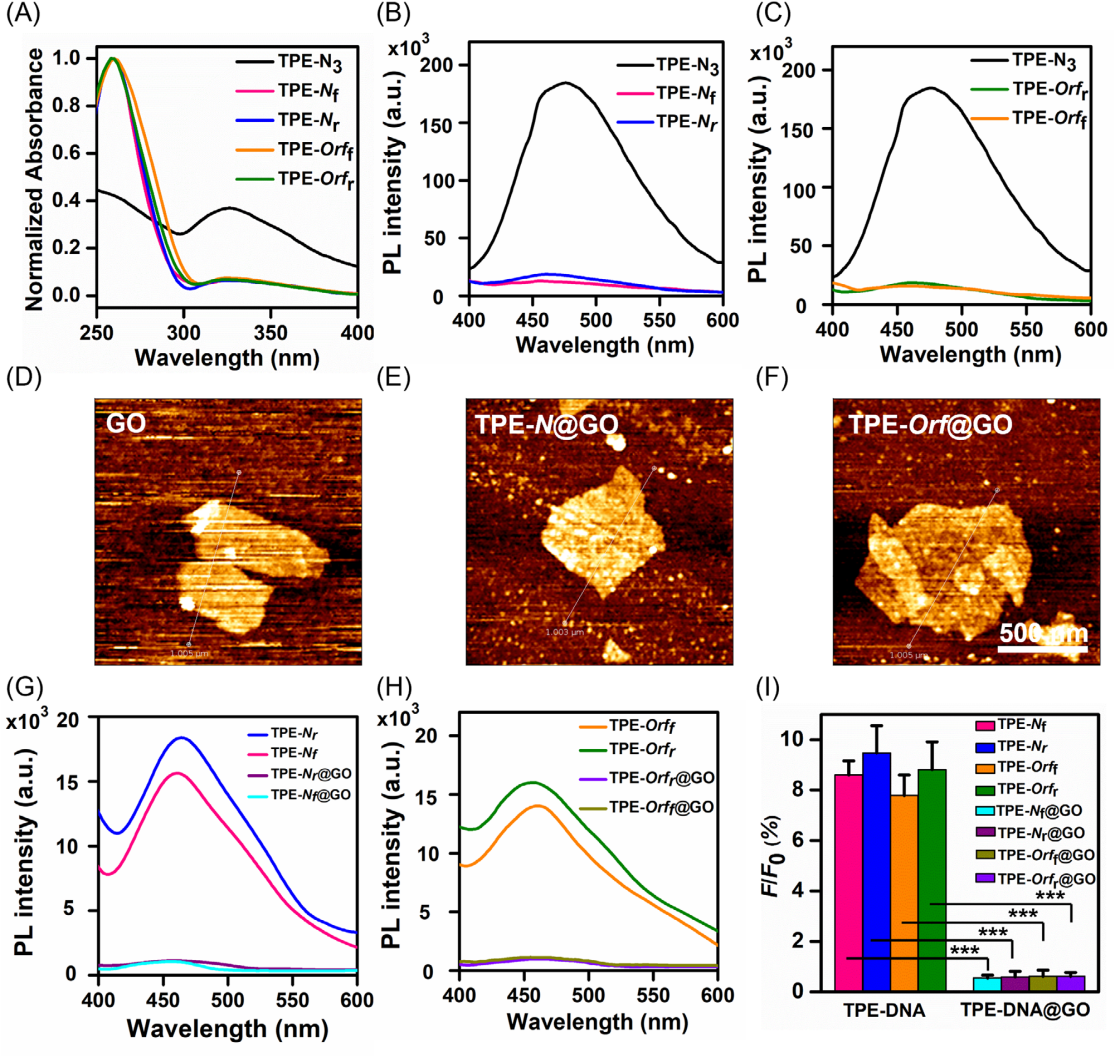
Physical property characterization of AIEgen and AIEgen@GO. (A) Ultraviolet‐visible (UV‐vis) absorbance of tetraphenylethene (TPE)‐N_3_ and TPE‐DNA. (B–C) Photoluminescence spectra of TPE‐N_3_, TPE‐*N*, and TPE‐*Orf*. (D–F) Atomic force microscopy images for examining the physical morphology of graphene oxide (GO), TPE‐*N*@GO, and TPE‐*Orf*@GO. (G–H) Photoluminescence spectra of TPE‐*N*, TPE‐*N*@GO, TPE‐*Orf*, and TPE‐*Orf*@GO. (i) Fluorescence intensity percentage of TPE‐DNA and TPE‐DNA@GO (*F*), which are normalized to the fluorescence intensity of TPE‐N_3_ (*F*_0_). Three independent measurements were performed, and the data are expressed as mean ± SD. Significant difference *p*‐value: ***<0.001. The concentrations of the TPE‐N_3_, TPE‐DNA, and TPE‐DNA@GO were 3 μM. All the spectra were measured in DMSO/H_2_O (v/v, 1/199). *λ*_ex_/*λ*_em_ = 320/458 nm

We synthesized the AIEgen, TPE‐DNA via the click reaction between azide‐functionalized TPE (TPE‐N_3_) and a specific 5′‐end alkyne‐modified single‐strand DNA primer (alkyne‐ssDNA).^[^ ^28^ ^]^ In this study, we chose two nucleic acid sequences, nucleoprotein (*N*) and replicate polyprotein open reading frame 1ab (*Orf1ab*) genes of SARS‐CoV‐2 virus as targets (Table S2). Accordingly, we designed and tested a total of two DNA‐primer pairs that have been validated for targeting *N* and *Orf1ab* genes of SARS‐CoV‐2 in qPCR, respectively (Figure 1A).^[^ ^41^ ^]^ Hence, one pair of the AIEgens is used to detect *N* sequence (forward primer‐TPE: TPE‐*N*_f_ and reverse primer‐TPE: TPE‐*N*_r_), and the other pair of the AIEgens is used to detect *Orf1ab* sequence (forward primer‐TPE: TPE‐*Orf*_f_ and reverse primer‐TPE: TPE‐*Orf*_r_) (Figure 1B). The synthesized AIEgens are then characterized by time‐of‐flight mass spectrometry. The mass spectrometry results indicated a significant increase of molecular weight (∼387 Da, corresponding to a TPE molecule) in the TPE‐DNA compared to both pairs of the free DNA primers, confirming the successful conjugation between alkyne‐ssDNA and TPE‐N_3_ (Figure S1). Ultraviolet‐visible (UV‐vis) spectroscopy showed that both TPE‐DNA and TPE‐N_3_ exhibited an absorption peak at 320 nm, attributing to π‐π* transition in TPE molecules.^[^ ^31^ ^]^ However, only TPE‐DNA showed a sharp peak at 260 nm, typically representing the presence of oligonucleotides (Figure 2A). More importantly, the AIEgen (TPE‐DNA) only occupied 8%–9% of the PL in TPE‐N_3_ molecules at the same concentration in dimethyl sulfoxide (DMSO)/H_2_O (v/v, 1/199) (Figure 2B,C). These results supported that the TPE‐DNA exhibited enhanced hydrophilicity because of the conjugation of DNA primers to TPE‐N_3_, while bare TPE‐N_3_ remained hydrophobic and aggregated in high water content solution to cause the AIE effect.^[^ ^32^, ^33^, ^42^ ^]^ Hence, we have successfully fabricated a low‐emissive water‐soluble AIEgen suitable for sensing target nucleic acid in an aqueous environment, such as body fluid.

We next coupled GO with the AIEgen forming AIEgen@GO (TPE‐*Orf*@GO and TPE‐*N*@GO) nanocomplex via π‐π stacking to minimize the background fluorescence of our platform (Figure 1B). The hydrodynamic size of AIEgen@GO was ∼30 nm larger than that of free GO (216 ± 3 nm) in phosphate buffer saline (PBS) measured by dynamic light scattering (Figure S2A). Also, the zeta potential of AIEgen@GO (e.g., TPE‐*Orf*_r_@GO: ‐42.4 ± 1.1 mV) was significantly more negative than the GO (−36.1 ± 1.8 mV) (Figure S2B), suggesting the inclusion of negatively charged oligonucleotides. In addition, our atomic force microscopy (AFM) images illustrated that the thickness of GO was 1.17 ± 0.18 nm, which increased ∼1 nm after TPE‐DNA adsorption onto GO (e.g., TPE‐*Orf*_r_@GO: 2.15 ± 0.25 nm, Figure 2D–F and Figure S3). The characterized results of the increased GO thicknesses are consistent with the previous findings of 2D materials physically absorbed with nucleic acids.^[^ ^43^, ^44^ ^]^ In short, our results indicated successful and stable adsorption between AIEgen and GO forming a reservoir of SARS‐CoV‐2 sensing probes.

To determine an optimal quenching efficiency (*Q*_e_) of our platform at the initial state, we incubated a fixed concentration of TPE‐DNA at 3 μM with GO concentrations varying from 5 to 200 μg ml^−1^. The PL spectrum of AIEgen@GO showed that the peak emission at 458 nm reduced gradually along with increased GO concentrations (Figure S4). The *Q*_e_ reached the maxima at ∼98% with 200 μg ml^−1^ of GO for both AIEgen pairs. To avoid over‐quenching during nucleic acids detection, we chose 150 μg ml^−1^ GO dispersion with *Q*_e_ at ∼93% to construct the AIEgen@GO for the rest of the detection study. Strikingly, the PL basal intensity of AIEgen@GO with *Q*_e_ ∼93% was only ∼0.6 % of the PL in TPE‐N_3_ and ∼8 % of the PL in the counterpart AIEgen at the same concentration (Figure 2G–I). Thus far, our findings specified the importance of integrating GO as an effective quencher into the AIEgen system to promote the signal‐to‐background ratio.

### 2.2 One‐step detection of SARS‐CoV‐2 complementary DNA (cDNA) sequence

We first examine the specificity and the limit of detection (LoD) of our AIEgen@GO via hybridizing the probe with ssDNA that mimic complementary DNA (cDNA) of the target *N* and *Orf1ab* sequences (*N*‐cDNA and *Orf1ab*‐cDNA) of varying concentrations (Figure S5). We select these two cDNAs because they typically derive from the reverse transcription of SARS‐CoV‐2 RNA collected from the body fluid of patients before performing a standard RT‐qPCR to confirm infection.^[^ ^8^ ^]^ We incubated the AIEgen@GO with the target cDNA at 37°C for 1 h to ensure a complete DNA hybridization before PL intensity measurement, which is more rapid than those assays using quantitative RT‐qPCR approaches for detection of the virus.^[^ ^5^ ^]^ In general, the single primer‐bearing AIEgen@GO (spAIEgen@GO: either TPE‐*N*_f_@GO or TPE‐*N*_r_@GO) at a fixed concentration showed that its peak fluorescent signal at 458 nm increased linearly along with the increase of *N*‐cDNA concentration (Figure S5C). Strikingly, their LoDs reached 200 pM, 10‐fold lower than those TPE‐*N* probes without GO, indicating that such AIEgen alone failed to sense a low concentration of target nucleic acids (Figure S5A–C). Similarly, we observe the same trend of *Orf1ab*‐cDNA detection as that of TPE‐*N*@GO by the spAIEgen@GO (either TPE‐*Orf*_f_@GO or TPE‐*Orf*_r_@GO) with LoD at 250 pM, which is also eight‐folds lower than those TPE‐*Orf* probes without GO (Figure S5D–F). To demonstrate the 2nd stage fluorescence recovery by RIR due to hybridization, we performed the detection experiment with only AIEgen and the target cDNA. As shown in Figure S6, both AIEgen probes (TPE‐*N*_r_ and TPE‐*Orf*_r_) showed a weak fluorescence signal before the addition of target *N*‐cDNA and *Orf1ab*‐cDNA. After adding target *N*‐cDNA, the fluorescence signals of AIEgen further enhanced from **“WEAK”** to **“STRONG”** due to RIR effects.^[^ ^32^, ^33^ ^]^

In order to prove that the fluorescent enhancement was caused by DNA hybridization between TPE‐DNA@GO nanoprobes with target cDNA instead of the degradation of DNA primer from TPE‐DNA in probes, agarose gel electrophoresis was performed before and after the administration of target cDNA. The results indicated that the probe‐cDNA complex groups (TPE‐*N*_f_@GO+*N*‐cDNA, TPE‐*N*_r_@GO+*N*‐cDNA, TPE‐*Orf*_f_@GO+*Orf1ab*‐cDNA, TPE‐*Orf*_r_@GO+*Orf1ab*‐cDNA) showed an obvious increase of base pair (bp) number compared with that of the target cDNA alone (∼50 bp for *N*‐cDNA and ∼70 bp for *Orf1ab*‐cDNA), demonstrating successful DNA hybridization between TPE‐DNA@GO nanoprobes with target cDNA (Figure S7A,B). In addition, we observe that the complex band exhibits brighter bands than that of the cDNA band without the addition of AIEgen@GO, suggesting a higher content of nucleic acids after DNA hybridization than those without hybridization (Figure S7A,B). Moreover, the nanodrop characterization results suggested that the incubation of AIEgen@GO probes in hybridization buffer without target cDNA at 37°C over 24 h did not significantly influence the DNA content (Figure S7C). These results agree with our postulation that TPE‐DNA probes exhibit a relatively high basal PL intensity, probably due to the pre‐existing of a weak RIR within TPE‐DNA molecules. Thus, the TPE molecules simultaneously strengthen RIR and recover the quenched fluorescent signal from GO via detection of target DNA, thereby exhibiting dual entities for maximizing the signal‐to‐background ratio.

**FIGURE 3:**
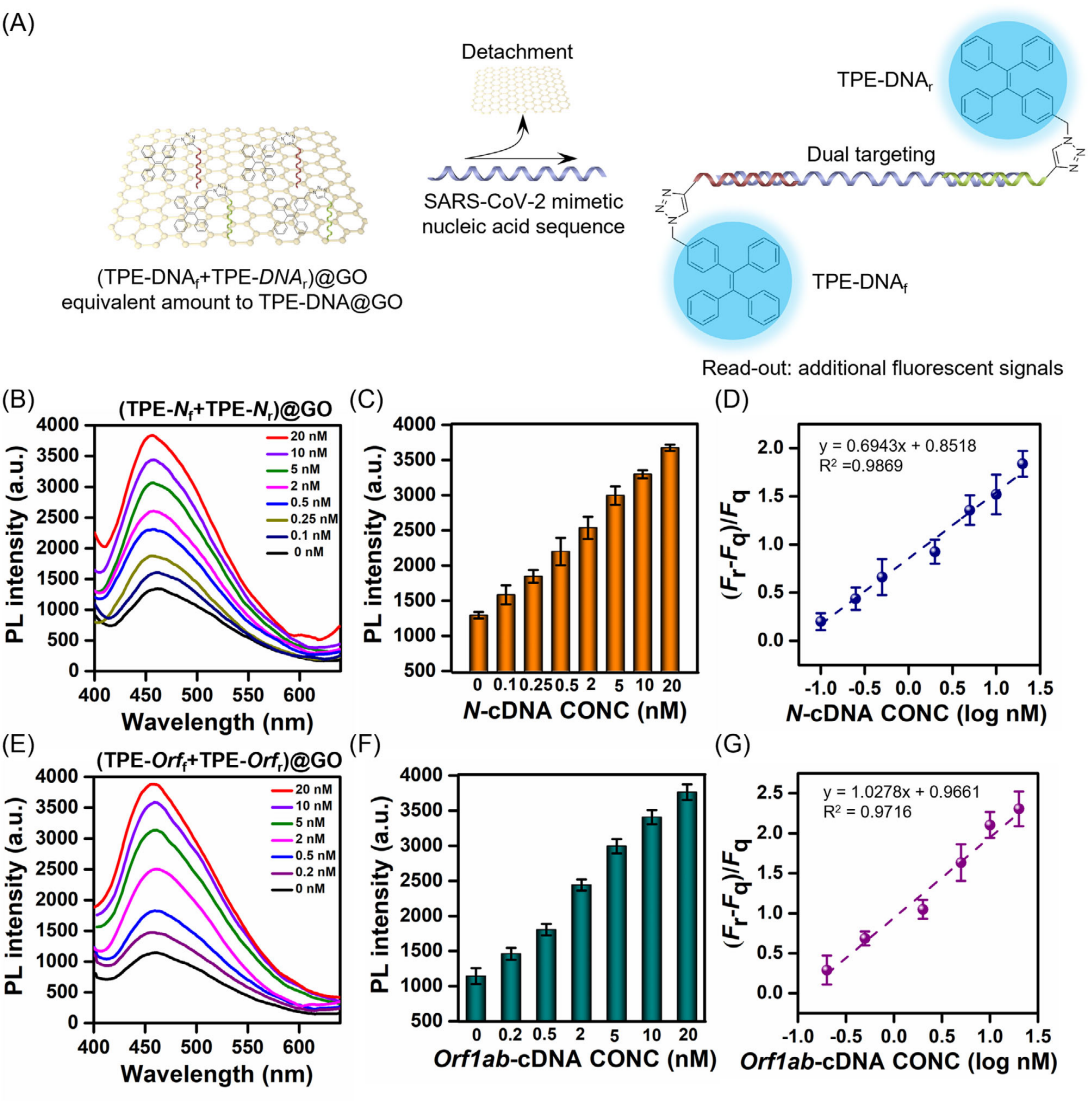
Signal enhancement via double‐sites DNA hybridization between a pair of primers‐bearing AIEgen@GO (ppAIEgen@GO) and the target sequence. (A) Schematic illustration of (tetraphenylethene [TPE]‐DNA_f_+TPE‐DNA_r_)@GO probing for two binding sites of Severe Acute Respiratory Syndrome Coronavirus 2 (SARS‐CoV‐2) mimetic DNA sequence. (B and E) Photoluminescence spectra of ppAIEgen@GO targeting *N*‐cDNA and *Orf1ab*‐cDNA, respectively, (C and F) with the illustration of peak PL intensity 458 nm. (D and G) Fitting logarithmic curve of ppTPE‐*N*@GO against the concentration of *N*‐cDNA and *Orf1ab*‐cDNA, respectively. Three independent measurements were performed, and the data are expressed as mean ± SD. The measured solution concentration was 3 μM. *λ*_ex_/*λ*_em_ = 320/458 nm

We suggest that GO can be a reservoir of delivering AIEgens that bear forward and reverse primers (e.g., TPE‐*N*_f_ and TPE‐*N*_r_), respectively, targeting the same nucleic acid sequence. Thus, we ask whether a pair of primers‐bearing AIEgen (ppAIEgen@GO: e.g., [TPE‐*N*_f_+TPE‐*N*_r_]@GO) with an equivalent amount (TPE molar ratio) to single primer bearing AIEgen (spAIEgen@GO: e.g., TPE‐*N*_f_@GO or TPE‐*N*_r_@GO) probing the same sequence can result in higher sensitivity (Figure 3A). Strikingly, the results showed that the LOD of ppAIEgen@GO for *N*‐cDNA reached 100 pM, two‐fold lower than that of using spAIEgen@GO probes (Figure 3B–D), potentially due to the doubling effect of DNA hybridization on the same target sequence for the signal amplification.^[^ ^45^ ^]^ Interestingly, the LOD of ppTPE‐*Orf*@GO was 200 pM, only 1.25‐fold lower than that of spTPE‐*Orf*@GO (Figure 3E,F). Such a slight increase of LOD was probably due to the larger nucleotide number of *Orf1ab*‐cDNA than *N*‐cDNA, which decreases the local density of the fluorescent signal.^[^ ^46^ ^]^ In addition, we confirmed that ppAIEgen@GO did not show cross‐reaction with scrambled cDNA sequence and remained a similar basal PL intensity after incubating with the scrambled cDNA (Figure S8). Altogether, the results demonstrate that our AIEgen@GO can simultaneously probe the dual segments of the same sequence for achieving an enhanced detection sensitivity.

**FIGURE 4:**
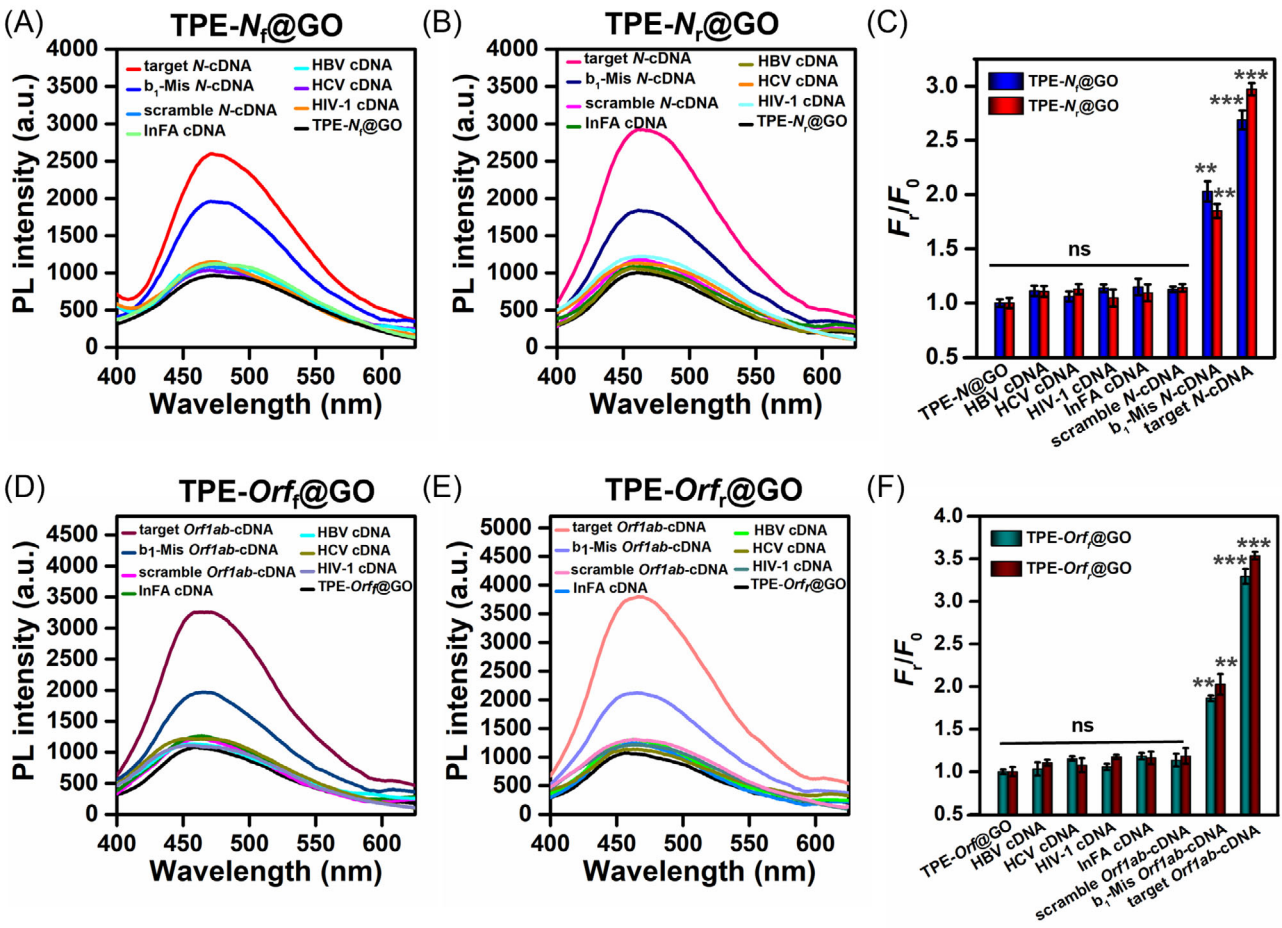
Photoluminescence spectra of (A) tetraphenylethene (TPE)‐*N*_f_@GO and (B) TPE‐*N*_r_@GO after incubating with target *N*‐cDNA, one base mismatch (b_1_‐Mis), scrambled *N*‐cDNA, InFA cDNA, HBV cDNA, hepatitis C virus (HCV) cDNA, and human immunodeficiency virus 1 (HIV‐1) cDNA. (C) Fluorescence intensity fold of (A) and (B) with emission at 458 nm. Photoluminescence spectra of (D) TPE‐*Orf*_f_@GO and (E) TPE‐*Orf*_r_@GO after incubating with target *Orf1ab*‐cDNA, one base mismatch (b_1_‐Mis), scrambled *Orf1ab*‐cDNA, InFA cDNA, HBV cDNA, HCV cDNA, and HIV‐1 cDNA. (F) Fluorescence intensity fold of (D) and (E) with emission at 458 nm, where *F*_r_ is the fluorescence intensity of probe after incubating with different ssDNA, and *F*_0_ presents the fluorescence intensity of probe without incubation, set as the control group in this experiment. The experiment was repeated three times, and the results are expressed as mean ± SD. Significant difference *p*‐value: *** <0.001, ** <0.01; n.s. means statistically nonsignificant. The concentrations of different ssDNA were 20 nM. The concentrations of TPE‐*N*_f_@GO, TPE‐*N*_r_@GO, TPE‐*Orf*_f_@GO, and TPE‐*Orf*_r_@GO were 3 μM. *λ*_ex_/*λ*_em_ = 320/458 nm

Furthermore, we assess the specificity of our AIEgen@GO toward detecting the two sequences of SARS‐CoV‐2 by hybridizing the corresponding probes with one base mismatch (b_1_‐Mis) and scrambled sequences of *N*‐cDNA and *Orf1ab*‐cDNA, respectively, as well as other types of viral cDNA sequences including influenza‐specific cDNA (InFA cDNA), hepatitis B virus (HBV), hepatitis C virus (HCV), and human immunodeficiency virus 1 (HIV‐1), as controls (Figure 4). The PL intensity of the AIEgen@GO incubating with the scrambled cDNA, or nonspecific viral cDNA sequences (InFA, HBV, HCV, and HIV‐1) at 20 nM (the highest concentration of the target cDNA used for the detection study) showed ∼3‐fold lower than the read‐outs of probing original target sequences (Figure 4C,F). Such resulting PL intensity is nearly weaker than the PL intensity at LoD by probing *N*‐cDNA or *Orf1ab*‐cDNA, suggesting that nonspecific nucleic acids minimally interfere with the AIEgen signal. The results of agarose gel electrophoresis exhibited no change of bp number between probe‐scrambled cDNA complex and scrambled cDNA alone (Figure S7A,B), indicating that AIEgen@GO probes did not interact with the scrambled cDNA sequences and the fluorescence recovery is really due to the specific hybridization. b_1_‐Mis cDNA (*N*‐cDNA or *Orf1ab*‐cDNA) enabled hybridization with AIEgen@GO probes with fluorescence recovery, but their emission intensities were almost 1.5‐fold lower than the intact target cDNA, indicating the high selectivity of our AIEgen@GO probes (Figure 4C,F). Meanwhile, the detection performance of AIEgen@GO probes toward *N*‐cDNA or *Orf1ab*‐cDNA was not disturbed by the presence of nonspecific virus cDNA (Figure S9). AIEgen@GO nanoprobes incubating with *N*‐cDNA or *Orf1ab*‐cDNA in the presence of one of the other nonspecific virus cDNAs (e.g., InFA, HBV, HCV, HIV‐1) resulted in signals similar to those in the absence of the nonspecific viral cDNA (Figure S9A–D). The following agarose gel electrophoresis confirmed that the detection performance of AIEgen@GO probes toward *N*‐cDNA or *Orf1ab*‐cDNA was not disturbed by the presence of nonspecific target DNA, InFA cDNA, as evidenced by the result of agarose gel electrophoresis (Figure S9E,F). Moreover, the lane containing a direct mixture of the probes (TPE‐*Orf*@GO or TPE‐*N*@GO) with InFA cDNA showed no significant shift of banding position from InFA cDNA, indicating that our biosensor minimally interacted with nonspecific nucleic acids. Furthermore, we explored the stability of our AIEgen@GO probes in multiple aspects. Firstly, the basal fluorescence of TPE‐*N*_f_@GO, TPE‐*N*_r_@GO, TPE‐*Orf*_f_@GO, and TPE‐*Orf*_r_@GO in PBS with varying pH (ranging from 6.5 to 8.0) and also different types of buffers (PBS, Tris‐EDTA buffer, borate buffered saline, bovine serum albumin solution, and cell culture medium) for 24 h did not exhibit significant fluctuation among these conditions (Figure S10A,B). Besides, these conditions also minimally influence the sensitivity performance of AIEgen@GO probes (Figure S10A,B). In addition, recovered fluorescence signals of AIEgen@GO probes incubating with corresponding target cDNA at 37°C reached maximum within 1 h and maintained a relatively constant intensity signal over 24 h (Figure S10C). Additionally, the detection performance of AIEgen@GO probes was evaluated in cell medium, emulating physiological body fluid. Intriguingly, the trend of fluorescent signal recovery of the AIEgen@GO in this condition was highly similar to that of those in the original incubation buffer (TE buffer) with the same LoD (Figure S11). Likewise, DMEM did not elevate the fluorescent signal even with the presence of scrambled cDNA and the AIEgen@GO probes, suggesting that our probes showed stable performance in physiological media. In short, these findings verified that our platform demonstrated a high sensitivity toward SARS‐CoV‐2 *N* and *Orf1ab* sequences and minimal cross‐reactivity against nonspecific or other common human coronavirus sequences.

### 2.3 Detection of SARS‐CoV‐2 RNA sequence

**FIGURE 5:**
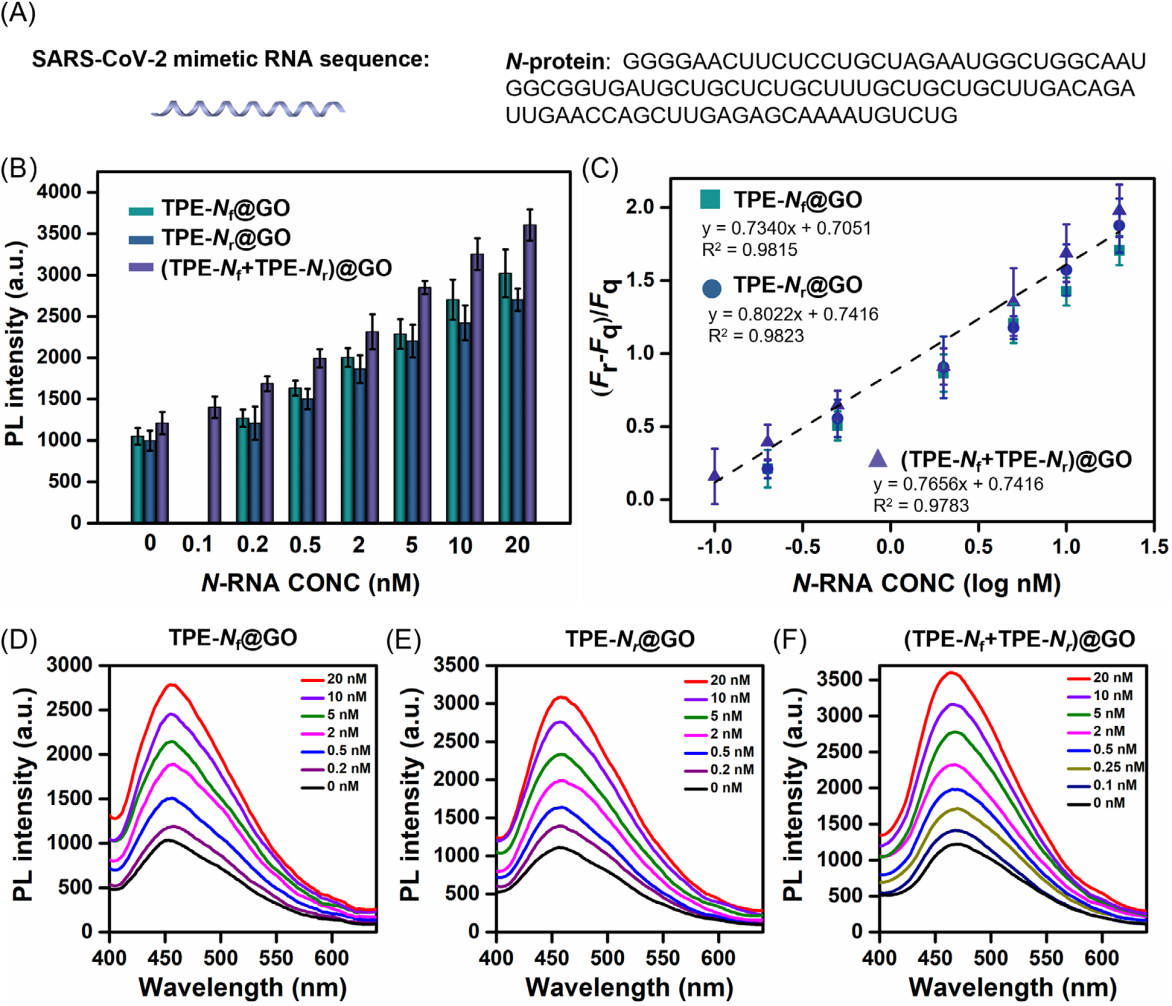
Confirmation of RNA detection ability by the AIEgen@GO platform. (A) Schematic illustration of Severe Acute Respiratory Syndrome Coronavirus 2 (SARS‐CoV‐2) mimetic *N*‐RNA sequence. (B) PL intensity of tetraphenylethene (TPE)‐*N*_f_@GO, TPE‐*N*_r_@GO, and (TPE‐*N*_f_+TPE‐*N*_r_)@GO with emission at 458 nm after incubating with *N*‐RNA and (C) respective fitting logarithmic curve after the addition of target *N*‐RNA with increasing concentration from 0.2 to 20 nM. Photoluminescence spectra of (D) TPE‐*N*_f_@GO, (E) TPE‐*N*_r_@GO, and (F) (TPE‐*N*_f_+TPE‐*N*_r_)@GO with the addition of target *N*‐RNA with different concentrations. The measured solution concentration was 3 μM. *λ*_ex_/*λ*_em_ = 320/458 nm

To further explore the capability of our AIEgen@GO probing RNA sequence, we select *N*‐RNA as the SARS‐CoV‐2 mimetic RNA sequence (Figure 5A).^[^ ^47^ ^]^ Consistent with the *N*‐cDNA detection results, the fluorescent signal intensity at 458 nm linearly increased along with increasing the concentration of *N*‐RNA (Figure 5B). Likewise, the LOD of spTPE‐*N*@GO (TPE‐*N*_f_@GO or TPE‐*N*_r_@GO) and ppTPE‐*N*@GO ((TPE‐*N*_f_+TPE‐*N*_r_)@GO) at 200 pM and 100 pM, respectively (Figure 5C–F). No significant fluorescence recovery was observed upon replacing the *N*‐RNA sequence with a scrambled RNA sequence, suggesting the excellent specificity of our probe (Figure S12). These results illustrated that our platform could detect both DNA and RNA with specific sequences. This ability is essential to indicate the presence of the SARS‐CoV‐2 RNA sequence in a sample without reverse transcription.

### 2.4 Rapid detection of SARS‐CoV‐2 plasmids with AIEgen@GO

**FIGURE 6:**
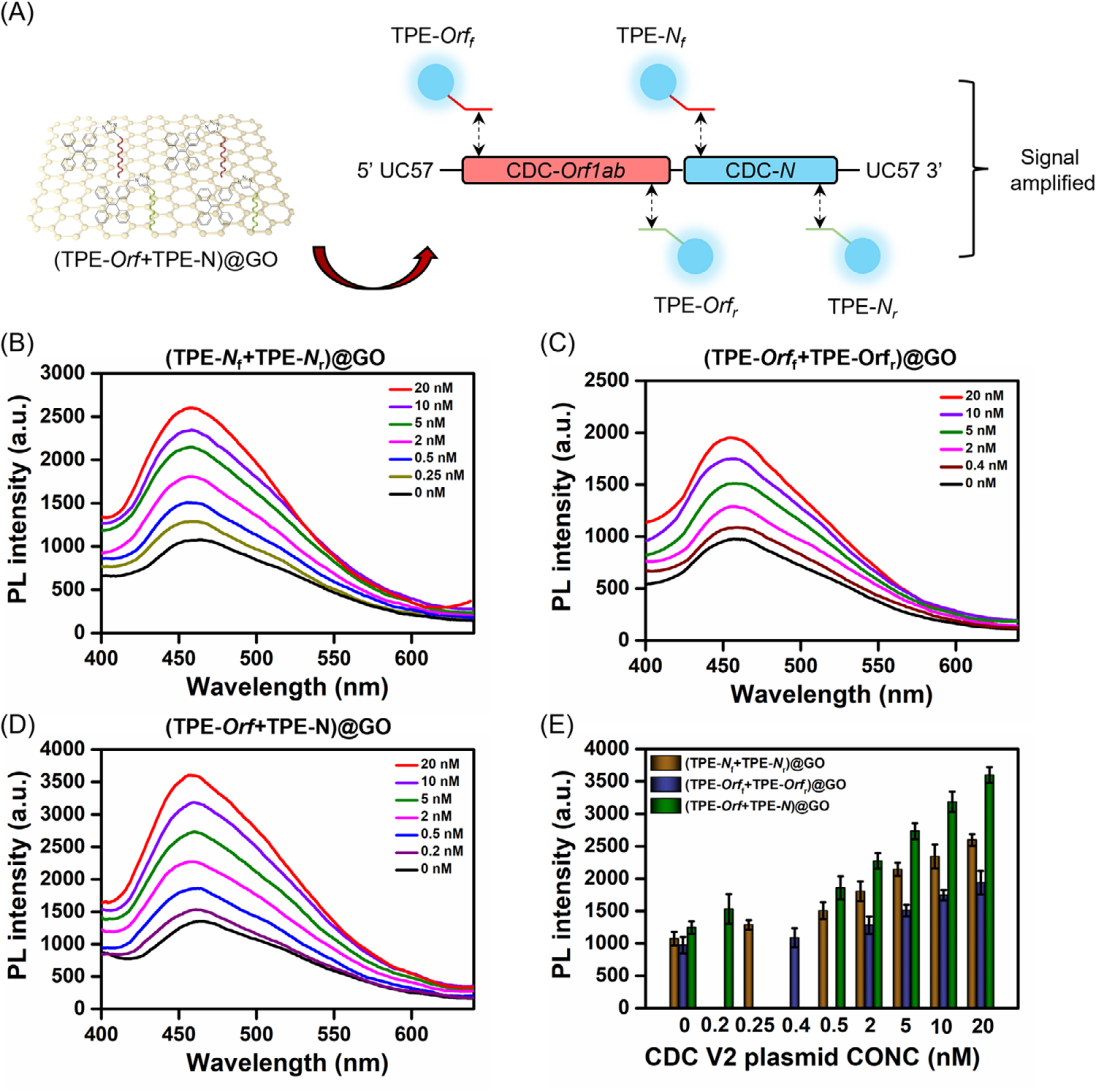
Detection of multiple target nucleic acid sequences from *N* and *Orf*‐bearing plasmid. (A) Schematic illustration of the ppAIEgen@GO probe for pUC57 CDC‐Severe Acute Respiratory Syndrome Coronavirus 2 (SARS‐CoV‐2) positive plasmid V2 (CDC‐V2 plasmid) detection. Photoluminescence spectra of (B) tetraphenylethene (TPE)‐*N*_f_+TPE‐*N*_r_@GO, (C) TPE‐*Orf*_f_+TPE‐*Orf*_r_@GO, (D) (TPE‐*Orf*+TPE‐*N*)@GO with the addition of denatured CDC‐V2 plasmid with different concentrations. The measured solution concentration was 3 μM. *λ*_ex_/*λ*_em_ = 320/458 nm, and (E) PL intensity of TPE‐*N*_f_+TPE‐*N*_r_@GO, TPE‐*Orf*_f_+TPE‐*Orf*_r_@GO, and (TPE‐*Orf*+TPE‐*N*)@GO with emission at 458 nm after incubating with CDC‐V2 plasmid

Here, CDC‐V2 plasmid comprising both *N* and *Orf1ab* sequence of the virus with pUC57 as the vector backbone was used to mimic real SARS‐CoV‐2 samples to evaluate the possible clinical screening capability. The US Centers for Disease Control and Prevention (CDC) has designed a standard RT‐qPCR assay and adopted a plasmid named CDC‐V2 as a positive control for SARS‐CoV‐2.^[^ ^41^ ^]^ CDC‐V2 plasmid comprises both *N* and *Orf1ab* sequence of the virus with pUC57 as the vector backbone (Table S2).^[^ ^38^ ^]^ With current instrumentation, the detection involves the addition of multiple components such as probes, primers, and amplification components and requires over 1∼2 h thermal cycles to complete the assay. Thus, we attempt to test if our AIEgen@GO can diagnose this positive control more cost‐effectively than the standard protocol (Figure 6A). The double‐strand DNA (dsDNA) of CDC‐V2 was first unwound at 98°C for 5 min to facilitate the hybridization with spAIEgen@GO, ppAIEgen@GO, or two different ppAIEgen@GO (total probes concentration is 3 μM) at 37°C for 1 h. The resulting PL intensity of spAIEgen@GO probing CDC‐V2 exhibited a similar pattern of probing the cDNA (Figure S13). Nevertheless, the LoD of the spAIEgen@GO detecting CDC‐V2 is 500 pM, one‐fold less sensitive than those probing the cDNAs (Figure S10), potentially due to the bulky plasmid body that increases the difficulty of hybridization between the AIEgen and the target sequence.^[^ ^48^ ^]^ Next, we examine the result of using ppAIEgen@GO (e.g., 1.5 μM TPE‐*N*_f_@GO+1.5 μM TPE‐*N*_r_@GO or 1.5 μM TPE‐*Orf*_f_@GO+1.5 μM TPE‐*Orf*_r_@GO) to probe CDC‐V2 (Figure 6B,C). Consistently, the resulting LoD reached 250 pM and 400 pM with ppTPE‐*N*@GO and ppTPE‐*Orf*@GO, respectively, a stronger detection signal than that of spAIEgen@GO probing CDC‐V2 at low concentration. We further evaluate the effect of utilizing two ppAIEgen@GO (forward and reverse primers of TPE‐*N*@GO and TPE‐*Orf*@GO) probing CDC‐V2, which has both *N* and *Orf1ab* sequence (Figure 6A). Intriguingly, this combinatorial detection enhanced the resulting LoD up to 200 pM (Figure 6D). The intact RNA sequence of SARS‐CoV‐2 should contain *N*, *Orf1ab*, and also other target sites.^[^ ^49^ ^]^ Hence, these findings outline our AIEgen@GO can simultaneously detect multiple target sites that can produce additional signals for lowering LoD to indicate the presence of SARS‐CoV‐2 nucleic acid sequences.

Currently, the gold standard for COVID‐19 diagnostics is RT‐qPCR, which has been utilized to detect SARS‐CoV‐2 viral load in clinical samples.^[^ ^50^, ^51^, ^52^ ^]^ The cycle threshold (Ct) value measured by RT‐qPCR is a clinical indicator of a detectable amount of SARS‐CoV‐2 virus in the RT‐qPCR experiment, which has an inverse relationship with the viral load.^[^ ^49^ ^]^ According to the literature, the Ct values of SARS‐CoV‐2 virus with *N* and *Orf1ab* as primary markers of SARS‐CoV‐2 in clinical samples ranged from around 10 to 37.^[^ ^39^, ^51^ ^]^ Similarly, we have also measured the Ct value of *N* and *Orf1ab* cDNA at LoD that is detectable by our sensors using RT‐qPCR. The Ct values detectable by our sensors were 19.60 ± 1.41 (*Orf1ab*‐cDNA at 200 pM) and 20.02 ± 1.19 (*N*‐cDNA at 100 pM), respectively (Table S3). Thus, our probes could potentially be used to detect clinical viral samples with Ct values ≤20. A comparison of our sensing platform with RT‐PCR technology for COVID‐19 diagnosis in terms of sensitivity, detection time, and cost is shown in Table S4. The sensitivity of our sensing platform is around Ct value of 20, which is lower than that of RT‐PCR above 30. This is understandable that RT‐PCR is an amplification‐based technique, and our technique is amplification‐free platform. In the future, the sensitivity of our platform can be further improved by combining our AIEgen@GO probes with isothermal amplification. The detection time of our platform is 1 h, which is much shorter than that of RT‐PCR around 6∼8 h. Moreover, our platform is low cost and does not need complicated and expensive RT‐PCR equipment. In short, our sensor is positioned as a rapid and amplification‐free nucleic acid test for the first screening of COVID‐19 in the clinical samples prior to PCR testing.

## 3 CONCLUSIONS

In summary, we demonstrate a simple, rapid, cost‐effective, and efficient AIEgen@GO‐based nano‐sensing platform for one‐step SARS‐CoV‐2 nucleic acid detection. We validate the importance of GO to suppress weak RIR‐induced background fluorescence of the AIE‐derivate sensor for yielding a high signal‐to‐background result for virus nucleic acids detection. Our findings support that our platform can detect multiple SARS‐CoV‐2 sequences in cDNA, RNA, and virus plasmids with high specificity and sensitivity at pM level without amplification, which can be a rapid POC residence community‐level nucleic acid testing for COVID‐19 as an initial screening. To further improve the sensitivity of AIEgen@GO platform, reverse transcription and recombinase polymerase isothermal amplification assay may be employed to amplify the copy number of target nucleic acids before the measurement in our future application. More importantly, the manufacturing process of our AIEgen only involves a single‐step click reaction and physical adsorption with the quencher (GO). Hence, this platform allows simply tailor‐made for a rapid genetic test of other pathogen nucleic acid sequences. We believe that this diagnostic strategy is highly promising for both infectious disease monitoring and translational application.

## 4 EXPERIMENTAL SECTION

### Materials

1‐((4‐azidomethyl) phenyl)‐1,2,2‐triphenylethene (TPE‐N_3_) were purchased from Alfa Chemical Co., Ltd (Zhengzhou, China) and used directly without further purification. Copper sulfate (CuSO_4_), DMSO, and Tris(3‐hydroxypropyltriazolylmethyl)amine (THPTA) were purchased from Sigma‐Aldrich Inc. PBS, Tris(hydroxymethyl)aminomethane (Tris‐HCl), Tris‐EDTA (TE)‐buffer, DNase/Rnase‐free ultrapure water, BlueJuice gel loading buffer, 10X Tris‐borate‐EDTA (TBE) buffer, Ultra Low Range DNA Ladder, and SYBR®Gold dye were purchased from ThermoFisher Scientific. DNase/RNase‐free ultrapure water was used throughout the experiments. All of the DNA and RNA sequence, CDC‐V2 plasmid, primers, and agarose powder were purchased from Sangon Biotech Co., Ltd (Shanghai, China). The information of these DNA and RNA sequences was listed in Table S2. Prior to experimentation, DNA and RNA were dissolved in TE buffer at a strand concentration of 100 μM and kept at −20°C. GO dispersion in water at a concentration of 2 mg ml^−1^ was purchased from XFNano Science and Technology Ltd. (Nanjing, China). RT‐qPCR was performed in a total volume of 25 μl using TB Green Premix Ex Taq Kit (TaKaRa Biotechnology, Dalian, China).

### Apparatus

The UV absorption spectra of TPE‐N_3_, TPE‐DNA (including TPE‐*N*_f_, TPE‐*N*_r_, TPE‐*Orf*_f,_ and TPE‐*Orf*_r_) were obtained by a UV‐vis‐NIR spectrophotometer (Ultrospec 2100 pro). Photoluminescence (PL) spectra of TPE‐N_3_, TPE‐DNA, and TPE‐DNA@GO, was measured by an Edinburgh F920 fluorescence spectrometer equipped with a xenon lamp source. The size distribution by intensity and zeta potential were measured with a Zetasizer Nanosystem (Malvern Instruments). The electrospray ionization (ESI) mass spectra of alkyne‐DNA and TPE‐DNA were acquired with an Agilent 6540 Liquid Chromatography‐ESI Quadrupole‐TOF Mass Spectrometer. The AFM images of GO and TPE‐DNA@GO were performed on a scanning probe microscope (NanoWizard AFM) under an ambient environment in peak force tapping mode. The agarose gels were visualized by using Chemi Doc system (Bio‐Rad). RT‐qPCR was carried out with a CFX96 Real‐Time PCR Detection System (Bio‐Rad).

### Preparation of TPE‐DNA@GO

TPE‐DNA was synthesized via copper‐catalyzed azide‐alkyne cycloaddition (CuAAC) reaction of azide group in TPE‐N_3_ with alkyne functionalized oligonucleotides (alkyne‐DNA, including alkyne‐*N*_f_, alkyne‐*N*_r_, alkyne‐*Orf*_f,_ and alkyne‐*Orf*_r_). Briefly, TPE‐N_3_ and alkyne‐DNA were mixed in 1 ml of DMSO/H_2_O (v/v, 1/1) at a final concentration of 1.5 μM and 1 μM, respectively. A freshly prepared aqueous solution of copper sulfate (100 μΜ, 10 μl) and copper ligand THPTA (200 μM, 10 μl) were premixed and then added to the above mixture in the former step, followed by adding fresh sodium ascorbate aqueous solution (200 μM, 10 μl). The reaction mixture was deoxygenated by bubbling with nitrogen gas for 30 min and then stirred for another 24 h at room temperature. The product was purified by dialysis against DMSO for 24 h using a dialysis bag with a membrane cutoff at 3 kDa to remove unreacted TPE‐N_3_ and then dialysis against deionized water for another 24 h to remove DMSO before lyophilization. Prior to use, GO dispersion was strongly sonicated for 1 h to yield a homogeneous aqueous solution. Lyophilized TPE‐DNA was dissolved in DMSO/H_2_O (v/v, 1/199) as a stock solution of 6 μM. For the quenching efficiency experiment, TPE‐DNA was incubated at a final concentration of 3 μM with GO dispersion (final concentration 5, 10, 20, 50, 100, 150, 200 μg ml^−1^) in PBS buffer for 30 min at room temperature. Then, the fluorescence intensity was determined by the spectrofluorometer, using a 2‐mm slit width with excitation at 320 nm. As regards the calculation of quenching efficiency (*Q*_e_), the fluorescence signal of 3 μM TPE‐DNA was set as control and labeled as *F*_0_. The quenching efficiency of GO was calculated according to the formula, *Q*_e_ (%) = (*F*_0_ – *F*_q_) / *F*_0_, where *F*_q_ represents the fluorescence intensity of TPE‐DNA after quenched by GO with various concentrations. All experiments were independently repeated three times (*n* = 3). In all detection experiments, TPE‐DNA@GO was prepared by incubating TPE‐DNA with GO dispersion in H_2_O to a final concentration of 3 μM and 150 μg ml^−1^, respectively. The obtained mixture was incubated for 30 min at room temperature and then utilized in a detection assay.

### Detection of SARS‐CoV‐2 DNA and RNA sequences

Throughout the detection experiments, TPE‐DNA@GO were incubated with target *N*‐cDNA, *Orf1ab*‐cDNA, and *N*‐RNA in DNase/RNase‐free TE buffer (10 mM Tris, 1 mM EDTA, 100 mM NaCl, 5 mM MgCl_2_). TPE‐DNA@GO was added into TE buffer containing a series of concentrations of *N*‐cDNA, *Orf1ab*‐cDNA, and *N*‐RNA (0∼20 nM). Briefly, a series of dilutions of the target cDNA solution in TE buffer were first prepared and then mixed with target probe solution for detection. For example, to detect *N*‐cDNA with final concentrations of 100 pM, 200 pM, 500 pM, 2 nM, 5 nM, 10 nM, 20 nM, the stock solution of *N*‐cDNA at 100 μM was diluted in TE buffer to serial concentrations of 200 pM, 400 pM, 1 nM, 4 nM, 10 nM, 20 nM, 40 nM. Then, an equal volume of TPE‐DNA@GO in H_2_O (6 μM, 100 μl) was mixed with different concentrations of target *N*‐cDNA prepared in the previous step. In this way, the concentration of TPE‐DNA@GO was fixed at 3 μM throughout the detection experiments to ensure comparability among different groups. Meanwhile, TE buffer with necessary ions was employed to provide favorable conditions for DNA/RNA hybridization process. Then, the mixture of TPE‐DNA@GO and target cDNA was incubated at 37°C for 1 h and protected from light before PL measurement. The sample preparation steps of *Orf1ab*‐cDNA detection were similar to that of *N*‐cDNA detection. To determine the specificity of TPE‐DNA@GO, controls were designed using one base mismatch (b_1_‐Mis) and scrambled sequences of *N*‐cDNA and *Orf1ab*‐cDNA, scrambled *N*‐RNA, and InFA‐cDNA. These control sequences were detected by TPE‐DNA@GO at the same concentration and with the same procedures as target cDNA and RNA sequences. After hybridization at 37°C for 1 h, fluorescence spectra of each sample were measured with excitation at 320 nm, and emission scanned from 400 nm to 600 nm. All measurement was performed with three independent samples.

### Detection of SARS‐CoV‐2 plasmids

CDC‐V2 plasmid was dissolved in TE buffer at a stock concentration of 100 μM. First, the CDC‐V2 plasmid was heated at 98°C in a water bath for 5 min and then immediately cooled on ice for a further 10 min. Then, TPE‐DNA@GO was incubated with different concentrations of denatured CDC‐V2 plasmid in TE buffer at 37°C for 1 h before PL measurement. Each experiment group was tested with three replicates.

### Agarose gel electrophoresis

AIEgen@GO probes (TPE‐*N*_f_@GO, TPE‐*N*_r_@GO, TPE‐*Orf*_f_@GO, and TPE‐*Orf*_r_@GO), target cDNA (*N*‐cDNA and *Orf1ab*‐cDNA), and probe‐cDNA complex (TPE‐*N*_f_@GO+*N*‐cDNA, TPE‐*N*r@GO+*N*‐cDNA, TPE‐*Orf*_f_@GO+*Orf1ab*‐cDNA, TPE‐*Orf*_r_@GO+*Orf1ab*‐cDNA) were analyzed on 5% agarose gel electrophoresis premixed with 0.1% SYBR®gold dye. Briefly, all the test samples were prepared by mixing the probes, cDNA, probe‐cDNA complex with BlueJuice gel loading buffer (v/v, 9/1) followed by loading on the gels. DNA ladder (size range: 10 bp ∼ 300 bp) in the same buffer was also loaded into the gel to assess the bp number of DNA fragments. Then, the agarose gels were run in 0.5X TBE buffer at a constant voltage (80 V) for 2 h for maximum separation. Finally, the agarose gels were observed under a UV transilluminator, and photographs were taken by a Chemi Doc system (Bio‐Rad).

### RT‐qPCR

The RT‐qPCR was performed with a TB Green Premix RT‐PCR Kit (Takara, Dalian, China) according to the protocol provided by the manufacturer. Briefly, *N*‐cDNA, *Orf1ab*‐cDNA, and CDC‐V2 plasmid were diluted in DNase/RNase‐free ultrapure water and then mixed with TB green dye and corresponding primers before PCR amplification on the CFX96 Real‐Time PCR detection system. CT values were calculated by the Real‐Time PCR Analysis Software (Bio‐Rad).

### Data analysis

The LOD of the sensing system was determined by the lowest observed effect concentrations, which was the lowest tested concentration significantly different from the control group by three times of standard derivation (*p* < 0.01).^[^ ^33^, ^53^ ^]^ In order to analyze LOD, TPE‐DNA@GO probes incubated with corresponding target cDNA solutions with different concentrations (2 pM, 20 pM, 50 pM, 100 pM, 200 pM, 500 pM, 2 nM, 5 nM, 10 nM, 20 nM) for 1 h at 37°C before PL measurement. Then, the fluorescence intensity at 458 nm of probes with or without target cDNA was compared by one‐way ANOVA followed by Dunnett's multiple comparison test via statistics software (IBM SPSS 26.0). The LOD of the probes is the lowest concentration, which is significantly higher than that of probes without the addition of target cDNA by three times of standard derivation (*p* < 0.01). In addition, the fitting of a linear relationship between relatively recovered fluorescence signal (*F*_r_ − *F*_q_)/*F*_q_ (*F*_q_: fluorescence intensity of probes, *F*_r_: fluorescence intensity probe‐cDNA complex) and logarithmic concentration of cDNA was analyzed by using Origin 9.0 software. The consequent correlation coefficient (R^2^) was employed to describe the linearity.

## CONFLICT OF INTEREST

The authors report no declaration of interest.

## AUTHOR CONTRIBUTIONS

Conceptualization, methodology, nanoprobe synthesis, and writing the original draft: *Qin Zhang*. Nanoprobe modification: *Bohan Yin*. Electronic microscopy experiment: *Linjie Ma*. Zeta potential measurement: *Yingying Huang*. Electronic microscopy experiment: *Xueying Shao*. Fluorescence spectrum measurement: *Chuanqi Li*. Electronic microscopy experiment: *Zhiqin Chu*. Writing‐review and editing: *Jianhua Hao*. Experiment design and writing‐review: *Siu Hong Dexter Wong*. Writing‐review and editing, supervision, and funding support: *Mo Yang*.

## Supporting information

Supporting information

## Data Availability

The data that support the findings of this study are available from the corresponding author upon reasonable request.
