# Supplementary material for: An AIEgen/graphene oxide nanocomposite (AIEgen@GO)‐based two‐stage “turn‐on” nucleic acid biosensor for rapid detection of SARS‐CoV‐2 viral sequence
Source: Aggregate (Hoboken). 2022 Apr 11:e195. Online ahead of print. doi: 10.1002/agt2.195 (PMC9073974; doi:10.1002/agt2.195)
Supplement: Supplementary file 1 — Supporting information [file AGT2-9999-0-s001.docx]

**Supporting Information**

**An AIEgen/graphene oxide nanocomposite (AIEgen@GO)-based two-stage “turn-on” nucleic acid biosensor for rapid detection of SARS-CoV-2 viral sequence**

Qin Zhang,^a^ Bohan Yin,^a^ Linjie Ma,^c^ Yingying Huang,^a^ Xueying Shao,^c^ Chuanqi Li,^a^ Zhiqin Chu,^c^ Changqing Yi,^d^ Jianhua Hao,^b^ Siu Hong Dexter Wong,^a,^* Mo Yang^a,^*

^a^Department of Biomedical Engineering, the Hong Kong Polytechnic University, Hong Kong 999077, China

^b^Department of Applied Physics, the Hong Kong Polytechnic University, Hong Kong 999077, China

^c^Department of Electrical and Electronic Engineering, Joint Appointment with School of Biomedical Sciences, the University of Hong Kong, Hong Kong 999077, China

^d^Key Laboratory of Sensing Technology and Biomedical Instruments (Guangdong Province), School of Biomedical Engineering, Sun Yat-Sen University, Guangzhou, 510006, P. R. China

***Corresponding Authors:**

Siu Hong Dexter Wong: shongwong@polyu.edu.hk

Mo Yang: mo.yang@polyu.edu.hk

| Probe Name | Sensing Principle | Analytes | LOD | Reference |
| --- | --- | --- | --- | --- |
| P1-DSAI-GO | Cationic AIE molecules (e.g., DSA, DSAI, DSAC_2_N) with good water solubility show weak fluorescence in the solutions but intense emission in their aggregate states, which were induced by integrating with target probing ssDNA or aptamers through electrostatic interaction. Then AIE/ssDNA complex was adsorbed on the GO surface to quench the fluorescence under the FRET mechanism. Once in the presence of analytes (target ssDNA or peptide), AIE/dsDNA detached from GO, and thus the fluorescence of complex was recovered (single-stage turn-on). | target ssDNA(T1),  thrombin protein | T1 ssDNA: linear range from 25 to 200 nM.  thrombin: 3 ng/mL | ^[34]^  Anal. Chem. 2014, 86, 298 |
| DSAC_2_N/C-Apt/GO |  | chloramphenicol | 1.26 pg/mL | ^[35]^  ACS Omega 2018, 3, 12886 |
| TTAPE-ssDNA-GO |  | complementary target ssDNA | 2.5 nM | ^[36]^  Acta Biomater. 2017, 50, 334 |
| DSA-DNA/GO |  | complementary target ssDNA | ~170 pM | ^[37]^  Small 2016, 12, 6613 |
| TPE-DNA@GO | Two-stage fluorescence recovery strategy: when TPE-ssDNA associates with targeted ssDNA to form a duplex-TPE molecule, the duplex dissociates from GO leading to fluorescence recovery. Moreover, the fluorescence will be further enhanced due to the partially RIR of the phenylene rings in the TPE structure, mainly resulting from the rigidity and mass change from ssDNA to dsDNA. | SARS-CoV-2 mimetic sequences (*N* and *Orf1ab* genes) | 100 pM for *N* gene (both ssDNA and RNA), 200 pM for *Orf1ab* gene (ssDNA) and CDC-V2 plasmid | This study |

Table S1. Comparison of fluorescent biosensors based on AIEgen-labelled oligonucleotide probes coupling with GO.

| Name | Sequence (5’ – 3’) |
| --- | --- |
| alkyne-*N*_f_ | HC≡C-ATTCTAGCAGGAGAAGTTCCCC |
| alkyne-*N*_r_ | HC≡C-CAGACATTTTGCTCTCAAGCTG |
| alkyne-*Orf*_f_ | HC≡C-TTAAGTGTAAAACCCACAGGG |
| alkyne-*Orf*_r_ | HC≡C-ACGATTGTGCATCAGCTGA |
| *N* primer Forward | GGGGAACTTCTCCTGCTAGAAT |
| *N* primer Reverse | CAGACATTTTGCTCTCAAGCTG |
| *Orf1ab* primer Forward | CCCTGTGGGTTTTACACTTAA |
| *Orf1ab* primer Reverse | ACGATTGTGCATCAGCTGA |
| *N*-cDNA | GGGGAACTTCTCCTGCTAGAATGGCTGGCAAT  GGCGGTGATGCTGCTCTTGCTTTGCTGCTGCTT  GACAGATTGAACCAGCTTGAGAGCAAAATGTCTG |
| *Orf1ab*-cDNA | CCCTGTGGGTTTTACACTTAAAAACACAGTCTGTA  CCGTCTGCGGTATGTGGAAAGGTTATGGCTGTAGT  TGTGATCAACTCCGCGAACCCATGCTTCAGTCAGCT  GATGCACAATCGT |
| *N*-RNA | GGGGAACUUCUCCUGCUAGAAUGGCUGGCAAUG  GCGGUGAUGCUGCUCUUGCUUUGCUGCUGCUUG  ACAGAUUGAACCAGCUUGAGAGCAAAAUGUCUG |
| One base mismatch  (b_1_-Mis) of *N*-cDNA | GGGCAACTTCTCCTGCTAGAATGGCTGGCAA  TGGCGGTGATGCTGCTCTTGCTTTGCTGCTGC  TTGACAGATTGAAC CAGCTTGACAGCAAAATGTCTG |
| One base mismatch  (b_1_-Mis) of *Orf1ab*-cDNA | CCGTGTGGGTTTTACACTTAAAAACACAG  TCTGTACCGTCTGCGGTATGTGGAAAGGTTA  TGGCTGTAGTTGTGATCAACTCCGCGAACCC  ATGCTTCAG TCAGCTGATGCAGAATCGT |
| Scrambled *N*-cDNA | GTTCCGTCGCTTCATACTGCTCATTCGAGCCAT  GGCGAAGACGGAGCGACAGTATTGGTTATGTAT  TAGAGTGGACGGCTTGCATTCTTGTGGACGGAC |
| Scrambled *Orf1ab*-cDNA | ATAGTAGCTCGTCCTCGAGGTGGCGTATTCGGAA  GATCGTCAATCTGTATTGACTGCTCCAACATTCCA  AGACGTCCTAACACTGTGTGCTTGAGGATTCCG CGGAACAATCTTGGTAT |
| Scrambled *N*-RNA | GUUCCGUCGCUUCAUACUGCUCAUUCGAGCCAUG  GCGAAGACGGAGCGACAGUAUUGGUUAUGUAUUAG  AGUGGACGGCUUGCAUUCUUGUGGACGGAC |
| HBV cDNA | CTGGATCCTGCGCGGGACGTCCTT |
| HCV cDNA | CACGCCCAAATCTCC |
| HIV-1 cDNA | GCTATACATTCTTACTATTTTATTTAATCCCAG |
| InFA cDNA | CGTGCCCAGTGAGCGAGGACTGCA |
| CDC-V2 plasmid | http://www.life-biotech.com/services/imgs/20200331pdf.pdf |

**Table S2.** The information of DNA and RNA sequences.


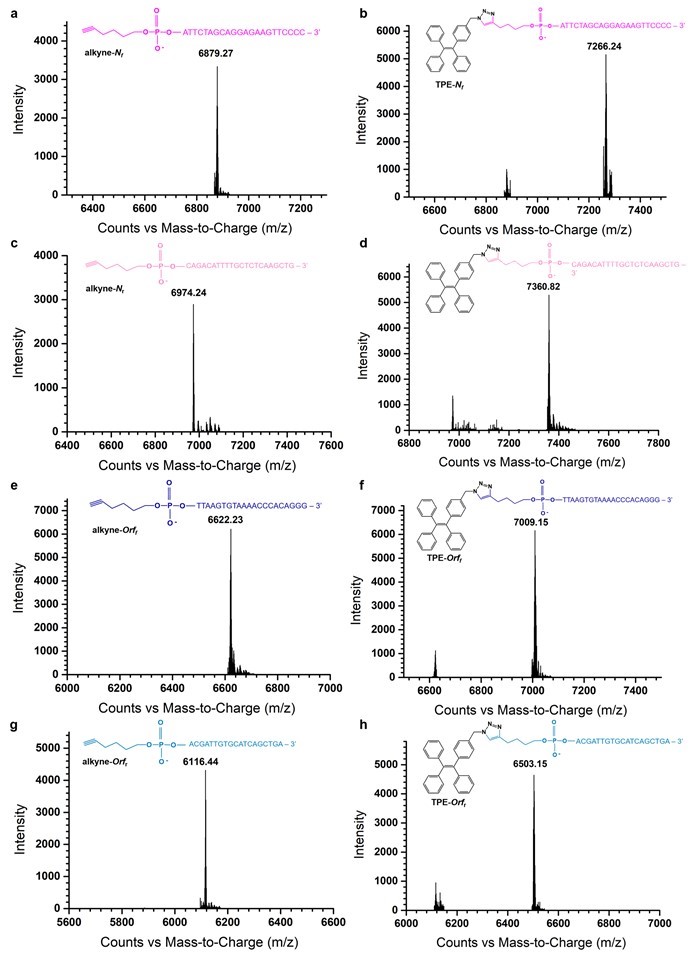


**FIGURE S1.** Electrospray ionization time-of-flight (ESI-TOF) mass spectrum of (a) alkyne-*N*_f_, (b) TPE-*N*_f_, (c) alkyne-*N*_r_, (d) TPE-*N*_r_, (e) alkyne-*Orf*_f_, (f) TPE-*Orf*_f_, (g) alkyne-*Orf*_r_, (h) TPE-*Orf*_r_.


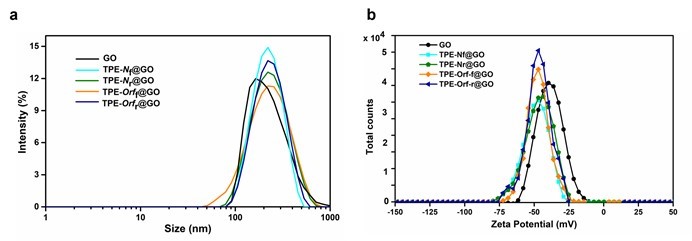


**FIGURE S2**. (a) Size distribution and (b) Zeta potential of GO, TPE-*N*_f_@GO, TPE-*N*_r_@GO, TPE-*Orf*_f_@GO, TPE-*Orf*_r_@GO.


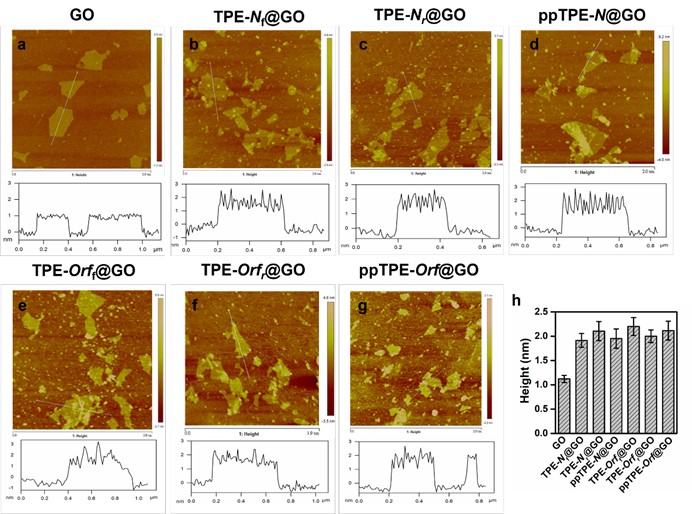


**FIGURE S3**. AFM images with height profiles of (a) GO, (b) TPE-*N*_f_@GO, (c) TPE-*N*_r_@GO, (d) ppTPE-*N*@GO, (e) TPE-*Orf*_f_@GO, (f) TPE-*Orf*_r_@GO, (g) ppTPE-*Orf*@GO. The bottom figures are height profiles along the lines in the upper AFM images. (h) Statistical distributions of the surface height. The results are expressed as mean ± SD. *n* = 3.

**
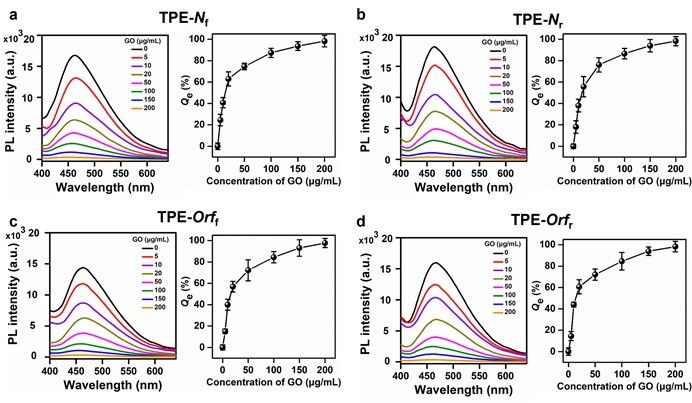
**

**FIGURE S4.** Photoluminescence spectra and quenching efficiency (*Q*_e_) of (a) TPE-*N*_f_, (b) TPE-*N*_r_, (c) TPE-*Orf*_f,_ and (d) TPE-*Orf*_r_ incubated with GO of a series of concentrations from 0 to 200 μg mL^-1^. The concentrations of TPE-*N*_f_, TPE-*N*_r_, TPE-*Orf*_f_ and TPE-*Orf*_r_ were all 3 μM. λ_ex_/λ_em_ = 320/458 nm.


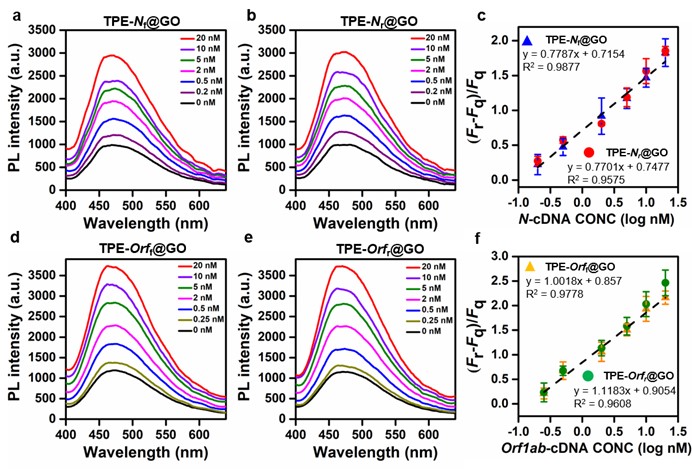


**FIGURE S5** Photoluminescence spectra of (a-b) TPE-*N*_f_@GO and TPE-*N*_r_@GO, and (d-e) TPE-*Orf*_f_@GO and TPE-*Orf*_r_@GO after incubating with target *N*-cDNA and *Orf1ab*-cDNA, respectively, at an increasing concentration from 0 nM to 20 nM. (c and f) Fitting logarithmic curve of the relative ﬂuorescence intensity of TPE-*N*_f_@GO (blue triangle), TPE-*N*_r_@GO (red circle), TPE-*Orf*_f_@GO (yellow triangle), and TPE-*Orf*_r_@GO (olive circle)*.* Three independent measurements were performed, and the data are expressed as mean ± SD. The measured solution concentration was 3 μM. λ_ex_/λ_em_ = 320/458 nm.


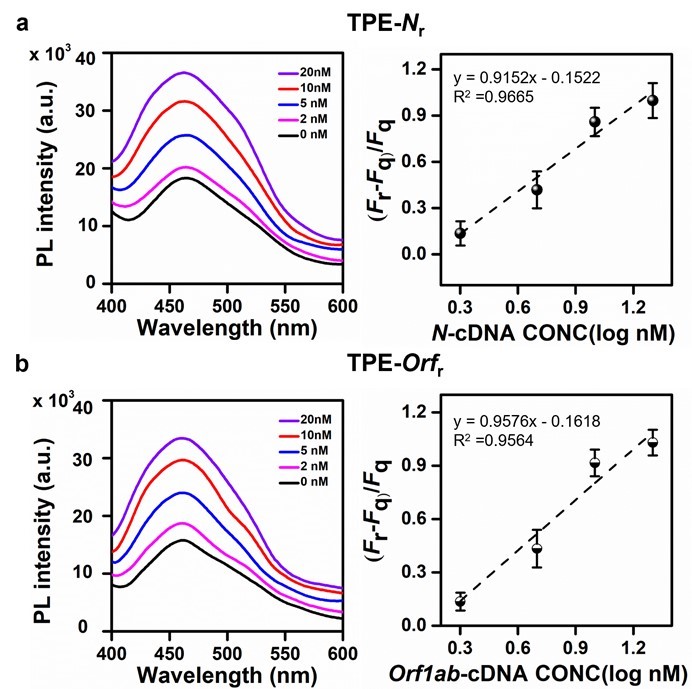


**FIGURE S6** Photoluminescence spectra and fitting curve of (a)TPE-*N*_r_ and (b) TPE-*Orf*_r_ after incubating with *N*-cDNA and *Orf1ab*-cDNA of a series of concentrations from 0 nM to 20 nM. The concentrations of TPE-*N*_r_ and TPE-*Orf*_r_ were 3 μM. λ_ex_/λ_em_ = 320/458 nm.

**
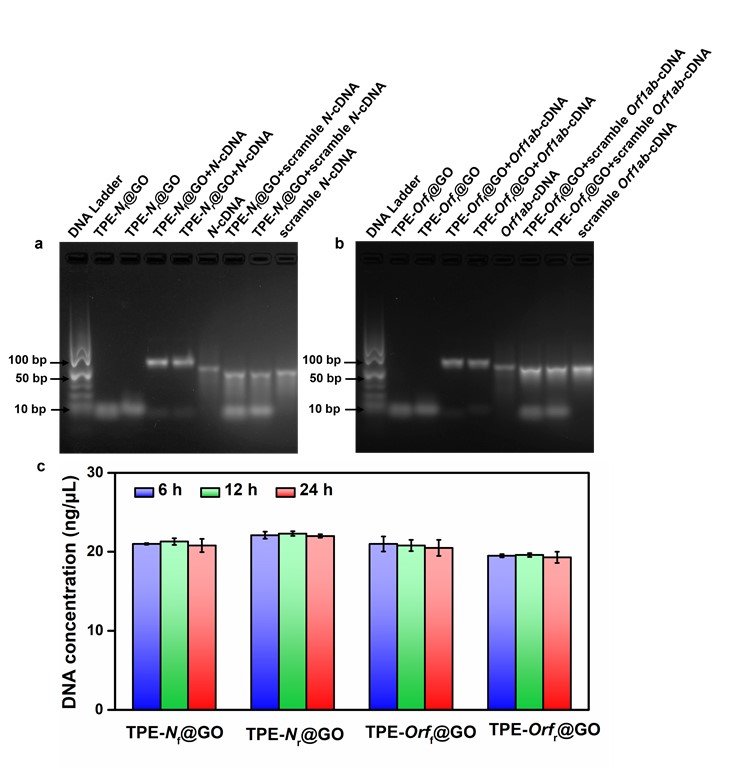
**

**FIGURE S7** Agarose gel electrophoresis of (a) TPE-*N*_f_@GO or TPE-*N*_r_@GO, (b) TPE-*Orf*_f_@GO or TPE-*Orf*_r_@GO after incubating with target cDNA or scramble cDNA. (c) DNA concentration of AIEgen@GO probes (TPE-*N*_f_@GO, TPE-*N*_r_@GO, TPE-*Orf*_f_@GO, and TPE-*Orf*_r_@GO) incubated in the hybridization buffer for different time before being measured by NanoDrop.


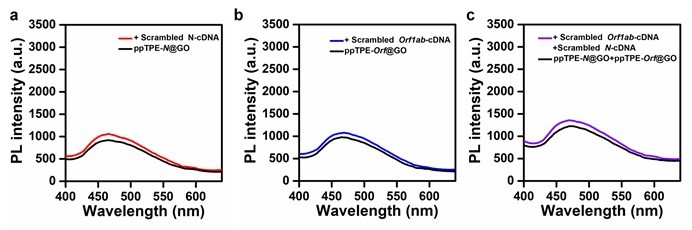


**FIGURE S8** Photoluminescence spectra of (a) ppTPE-*N*@GO, (b) ppTPE-*Orf*@GO and (c) ppTPE-DNA@GO (TPE-*N*@GO+TPE-*Orf*@GO) after incubating with scrambled *N*-cDNA, scrambled *Orf1ab*-cDNA, scrambled *N*-cDNA + scrambled *Orf1ab*-cDNA. The concentrations of ppTPE-*N*@GO, ppTPE-*Orf*@GO, and ppTPE-DNA@GO were 3 μM. The concentrations of scrambled *N*-cDNA and scrambled *Orf1ab*-cDNA were 20 nM. λ_ex_/λ_em_ = 320/458 nm.


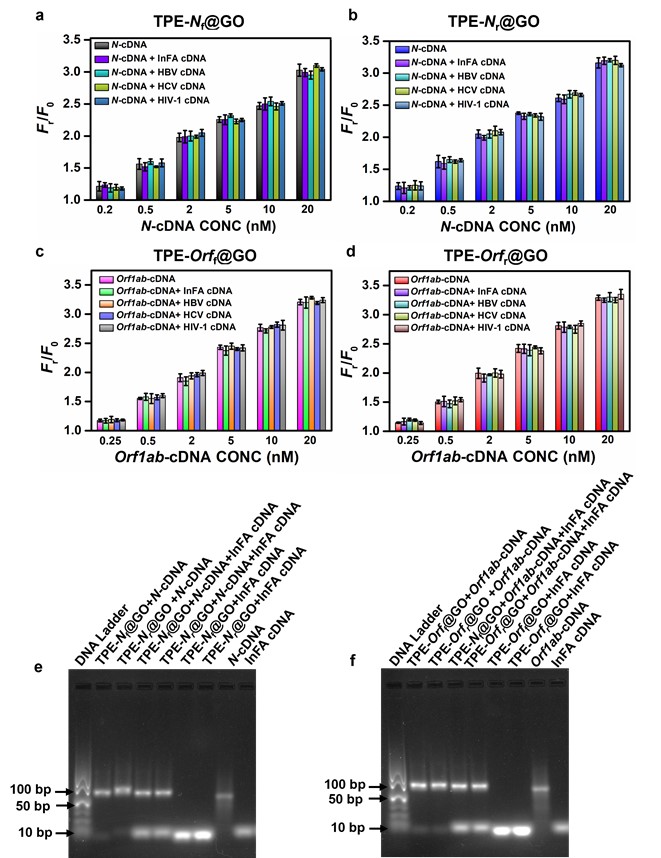


**FIGURE S9** Detection of target cDNA for SARS-CoV-2 in the presence of non-specific viral cDNA. Fluorescence intensity fold of (a) TPE-*N*_f_@GO, (b) TPE-*N*_r_@GO, (c) TPE-*Orf*_f_@GO, and (d) TPE-*Orf*_r_@GO after incubating with corresponding target cDNA with or without non-specific viral cDNA. Agarose gel electrophoresis of (e) TPE-*N*_f_@GO or TPE-*N*_r_@GO and (f) TPE-*Orf*_f_@GO or TPE-*Orf*_r_@GO probes after incubating with corresponding target cDNA with or without InFA cDNA.


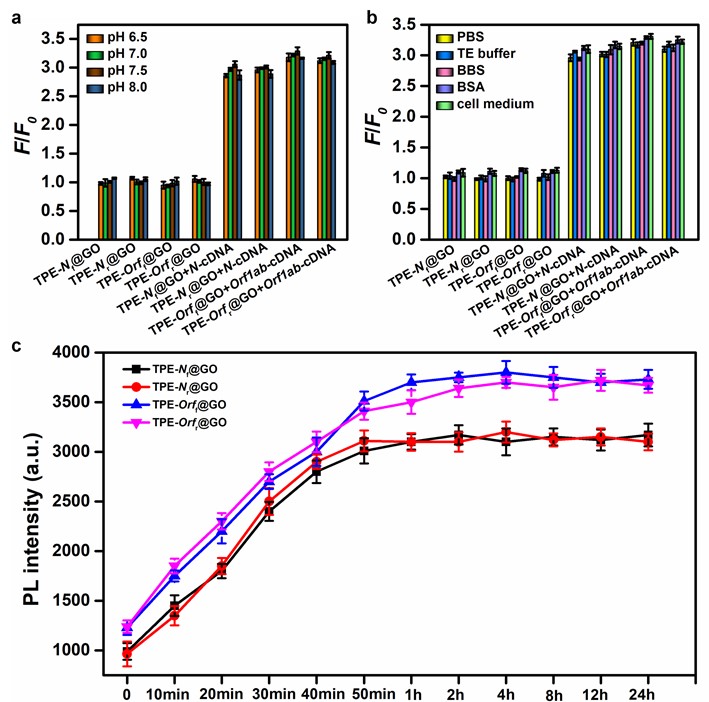


**FIGURE S10** The stability of the AIEgen@GO nanoprobes. The fluorescence intensity changes of TPE-*N*_f_@GO, TPE-*N*_r_@GO, TPE-*Orf*_f_@GO, and TPE-*Orf*_r_@GO dissolved in (a) PBS buffer with different pH values or (b) different buffers (PBS, TE buffer, borate buffered saline (BBS), bovine serum albumin (BSA), and cell medium) for 24 h and then incubated with corresponding target cDNA for an additional 24 h. (c) Fluorescence intensity of TPE-*N*_f_@GO, TPE-*N*_r_@GO, TPE-*Orf*_f_@GO, and TPE-*Orf*_r_@GO after incubating with corresponding target *N*-cDNA and *Orf1ab*-cDNA at 37 ℃ for different time.


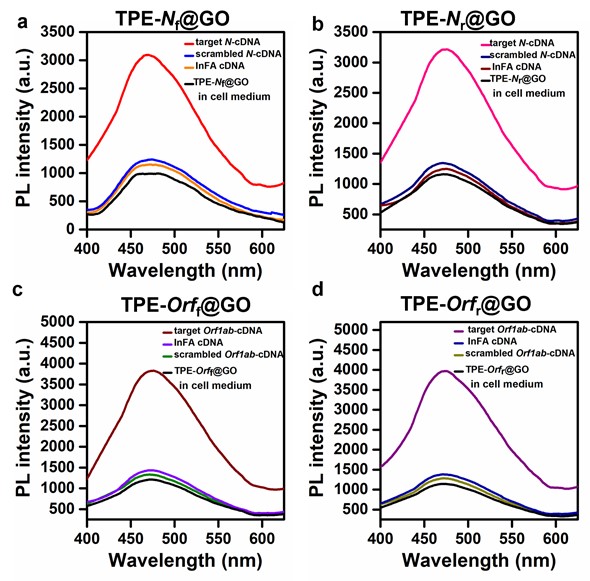


**FIGURE S11** Photoluminescence spectra of (a) TPE-*N*_f_@GO and (b) TPE-*N*_r_@GO after incubating with InFA cDNA, scrambled *N*-cDNA, and target *N*-cDNA in cell medium (DMEM). Photoluminescence spectra of (c) TPE-*Orf*_f_@GO and (d) TPE-*Orf*_r_@GO after incubating with InFA cDNA, scrambled *Orf1ab*-cDNA, and target *Orf1ab*-cDNA in DMEM. The concentrations of TPE-*N*_f_@GO, TPE-*N*_r_@GO, TPE-*Orf*_f_@GO, TPE-*Orf*_r_@GO were 3 μM. The concentrations of InFA cDNA, scrambled and target cDNA were 20 nM. λ_ex_/λ_em_ = 320/458 nm.

**
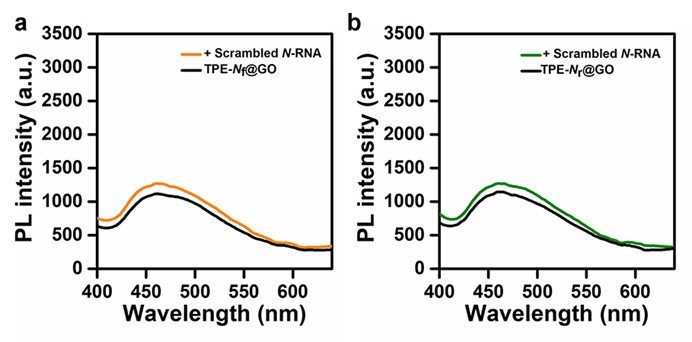
**

**FIGURE S12.** Photoluminescence spectra of (a) TPE-*N*_f_@GO and (b) TPE-*N*_r_@GO after incubating with scrambled *N*-RNA. The concentrations of TPE-*N*_f_@GO and TPE-*N*_r_@GO were 3 μM. The concentration of scrambled *N*-RNA was 20 nM. λ_ex_/λ_em_ = 320/458 nm.


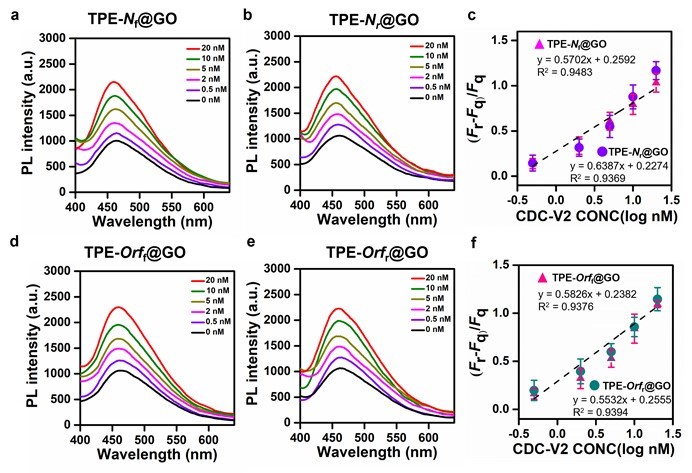


**FIGURE S13.** Photoluminescence spectra of (a) TPE-*N*_f_@GO and (b) TPE-*N*_r_@GO after incubating with denatured CDC-V2 plasmid of various concentrations. (c) The fitting logarithmic curve of TPE-*N*_f_@GO and TPE-*N*_r_@GO with the addition of denatured CDC-V2 plasmid. Photoluminescence spectra of (d) TPE-*Orf*_f_@GO and (e) TPE-*Orf_r_*@GO after incubating with denatured CDC-V2 plasmid of varying concentrations. (f) ﬁtting logarithmic curve of TPE-*Orf*_f_@GO and TPE-*Orf_r_*@GO with the addition of denatured CDC-V2 plasmid. The concentration of TPE-*N*_f_@GO, TPE-*N*_r_@GO, TPE-*Orf*_f_@GO, and TPE-*Orf*_r_@GO was 3 μM. λ_ex_/λ_em_ = 320/458 nm.

| Amplified genes | Sample source | Ct value | Ct range | Reference |
| --- | --- | --- | --- | --- |
| *N* gene + *Orf1ab* | swabs from SARS-COV-2 positive patients | median:26.4 | 9.2~37.8 | ^[51]^  Elife 2021, 10, 64683 |
| *N* gene + *Orf1ab* | nasopharyngeal swabs from SARS-COV-2 positive patients | / | *11~37* | ^[39]^  Nat. Biomed. Eng. 2020, 4, 1140 |
| *Orf1ab*-cDNA  (200 pM) | synthetic cDNA | mean±SD:  19.60±1.41 | / | This study |
| *N*-cDNA  (100 pM) | synthetic cDNA | mean±SD:  20.02±1.19 | / | This study |
| CDC-V2 plasmid  (200 pM) | synthetic cDNA | mean±SD:  16.09±2.12 | / | This study |

**Table S3.** Distribution of Ct value of *Orf1ab* and *N* gene in SARS-CoV-2 viral specimens, synthetic *Orf1ab*-cDNA and *N*-cDNA quantified by RT-qPCR.

| Parameters | AIEgen@GO probes | RT-PCR |
| --- | --- | --- |
| Sensitivity | Picomolar level  (Ct ~20) | ~100 copies/mL  (Ct~30) |
| Detection time | ~1 h | 6~8 h |
| Cost | Cost-effective  i) low cost probe and buffer (i.e., TE buffer)  ii) Fluorescence detector | Expensive  i) High cost PCR kits  ii) Expensive RT-PCR equipment |
| Experimental procedures | Easy operation | Need well-trained operators |

Table S4. Comparison of AIEgen@GO probes with RT-PCR for SARS-CoV-2 detection.
